# Characterization of molecular and cellular phenotypes associated with a heterozygous *CNTNAP2* deletion using patient-derived hiPSC neural cells

**DOI:** 10.1038/npjschz.2015.19

**Published:** 2015-06-24

**Authors:** Inkyu S Lee, Claudia M B Carvalho, Panagiotis Douvaras, Seok-Man Ho, Brigham J Hartley, Luciana W Zuccherato, Ian G Ladran, Arthur J Siegel, Shane McCarthy, Dheeraj Malhotra, Jonathan Sebat, Judith Rapoport, Valentina Fossati, James R Lupski, Deborah L Levy, Kristen J Brennand

**Affiliations:** 1 Departments of Psychiatry and Neuroscience, Icahn School of Medicine at Mount Sinai, New York, NY, USA; 2 Department of Molecular and Human Genetics, Baylor College of Medicine, Houston, TX, USA; 3 The New York Stem Cell Foundation, New York, NY, USA; 4 Internal Medicine Department, McLean Hospital, Belmont, MA, USA; 5 Cold Spring Harbor Laboratory, Cold Spring Harbor, NY, USA; 6 F. Hoffmann-La Roche Ltd, Basel, Switzerland; 7 Department of Psychiatry and Department of Cellular and Molecular Medicine, University of California San Diego, La Jolla, CA, USA; 8 Childhood Psychiatry Branch, National Institute of Mental Health, National Institutes of Health, Bethesda, MD, USA; 9 Human Genome Sequencing Center, Baylor College of Medicine, Houston, TX, USA; 10 Texas Children’s Hospital, Houston, TX, USA; 11 Department of Pediatrics, Baylor College of Medicine, Houston, TX, USA; 12 Psychology Research Laboratory, McLean Hospital, Belmont, MA, USA

## Abstract

Neurodevelopmental disorders, such as autism spectrum disorders and schizophrenia, are complex disorders with a high degree of heritability. Genetic studies have identified several candidate genes associated with these disorders, including contactin-associated protein-like 2 (*CNTNAP2*). Traditionally, in animal models or *in vitro*, *CNTNAP2* has been studied by genetic deletion or transcriptional knockdown, which reduces the expression of the entire gene; however, it remains unclear whether the mutations identified in clinical settings are sufficient to alter *CNTNAP2* expression in human neurons. Here, using human induced pluripotent stem cells (hiPSCs) derived from two individuals with a large (289 kb) heterozygous deletion in *CNTNAP2* (affecting exons 14–15) and discordant clinical outcomes, we have characterized *CNTNAP2* expression patterns in hiPSC neural progenitor cells, two independent populations of hiPSC-derived neurons and hiPSC-derived oligodendrocyte precursor cells. First, we observed exon-specific changes in *CNTNAP2* expression in both carriers; although the expression of exons 14–15 is significantly decreased, the expression of other exons is upregulated. Second, we observed significant differences in patterns of allele-specific expression in *CNTNAP2* carriers that were consistent with the clinical outcome. Third, we observed a robust neural migration phenotype that correlated with diagnosis and exon- and allele-specific *CNTNAP2* expression patterns, but not with genotype. In all, our data highlight the importance of considering the nature, location, and regulation of mutated alleles when attempting to connect genome wide association studies to gene function.

Structural variants and single-nucleotide variants involving Contactin-associated protein-like 2 (*CNTNAP2*) have been implicated in neurodevelopmental disorders, such as autism spectrum disorders, schizophrenia (SZ), epilepsy, language disorders, and cognitive impairments,^1^ but the relative risk associated with heterozygous mutations is unresolved.^2^ CNTNAP2 protein functions in axon guidance, dendritic arborization, spine development, and organization of myelinated axons (reviewed in ref. 1); complete loss of *Cntnap2* results in impaired migration of cortical projection neurons, reduced GABAergic neurons, and decreased neural synchrony in mice.^3^

Here, using human induced pluripotent stem cells (hiPSCs) derived from two related individuals with a large (289.3 kb) and heterozygous deletion in *CNTNAP2* and discordant clinical phenotypes, we have characterized *CNTNAP2* expression patterns in hiPSC neural progenitor cells (NPCs), two independent populations of hiPSC-derived neurons, and hiPSC-derived oligodendrocyte precursor cells (OPCs). Fibroblast samples were obtained from a female proband (DL7078), who met DSM-IV criteria for a diagnosis of schizo-affective disorder (depressed subtype) (SZ), and both parents (DL8735, DL5535); the proband and her clinically unaffected father are carriers (Figure 1a and Supplementary methods). The *CNTNAP2* deletion was initially identified in patient lymphocytes using the Nimblegen HD 2 platform and was subsequently independently confirmed using a high-density custom-designed Agilent array comparative genomic hybridization in DNA samples derived from individual leucocytes, Epstein–Barr virus-transformed lymphoblastoid cell lines, and fibroblasts (Figure 1b). Long-range PCR and Sanger sequencing narrowed down deletion breakpoint junctions; these map to introns, leading to loss of exons 14–15 in the affected allele (Figure 1c).

Non-integrating sendai viral reprogramming methods were used to generate three hiPSC lines from each member of the trio, as well as one hiPSC line each from five unrelated psychiatrically healthy controls with no DSM-IV diagnosis. All hiPSC lines were validated by long-term expansion beyond 10 passages, immunohistochemistry for pluripotency markers (Figure 1d, top), and normal karyotype (data not shown). Except where otherwise noted, experiments represent averaged results from three hiPSC lines each from the non-carrier Mother^+/+^, the unaffected carrier Father^+/−^, and the SZ Daughter^+/−^, as well as one hiPSC line from each of five ethnicity-matched unrelated controls (three males; two females). hiPSCs were differentiated by dual SMAD inhibition^4^ of embryoid bodies to yield neural rosettes, which were subsequently expanded as NPCs^5^ (Figure 1d, middle); neurons were generated by either 6 weeks of directed differentiation to a forebrain neuronal fate^5,6^ or rapid 2-week lentiviral *Ngn2* induction to glutamatergic neurons^7^ (Figure 1d, bottom).

*CNTNAP2* has eight transcript variants; the full-length transcript is comprised of 24 exons (NM_014141). We performed a series of qPCR experiments to determine exon-specific and allelic-specific expression differences due to the presence of the deletion (Figure 2a). Full-length *CNTNAP2* expression was low in fibroblasts (Figure 2b) and hiPSCs (Figure 2c). In NPCs, the SZ Daughter^+/−^ and unaffected carrier Father^+/−^ showed a downward trend in expression of deleted exons 14–15; unexpectedly, we detected significantly increased expression of exons 23–24 in both carriers, SZ Daughter^+/−^ (*P*=0.0185) and Father^+/−^ (*P*=0.0190) (Figure 2d), suggesting that the presence of the deletion may alter the transcript expression in NPCs. Interestingly, in hiPSC-derived 6-week-old forebrain neurons, we also observed a significantly increased *CNTNAP2* expression of exons 2–3 (*P*=0.0016) and exons 23–24 (*P*=0.0030) only in the SZ proband (Figure 2e), again suggesting that the deletion leads to increased full-length transcript expression in a phenotype-specific way. Finally, in 2-week-old *Ngn2*-induced neurons, in a population that had reached electrophysiological maturity, we observed significantly decreased expression of the deleted exons 14–15 (*P*=0.0387) in the SZ Daughter^+/−^ only, relative to five unrelated controls (Figure 2f).

We assayed neuronal allele-specific expression of *CNTNAP2* from the unaffected and deleted alleles by qPCR with primers targeting exons 13–16 on *Ngn2*-induced neurons; amplified *CNTNAP2* cDNA is either a 517 bp (wild type) or a 232 bp (deletion) amplicon. Expression from the intact allele (517 bp) was decreased and the deleted allele (232 bp) was increased in SZ Daughter^+/−^, relative to unrelated controls; the unaffected carrier Father^+/−^ showed expression from both the intact allele (517 bp) and the deleted allele (232 bp), albeit at lower levels (Figure 2g).

We generated OPCs from hiPSCs,^8^ and analyzed cultures at day 64, comprised of ~50% O4^+^ late OPCs (15% O4^+^/MBP^+^ mature oligodendrocytes) as well as ~15% astrocytes and ~20% neurons (Figure 2h). Expression of deleted exons 14–15 was significantly decreased in both the SZ Daughter^+/−^ (*P*<0.0001) and the unaffected carrier Father^+/−^ (*P*<0.0001) relative to one unrelated control (Figure 2i). Here too, the SZ Daughter^+/−^ expressed predominantly the mutant allele, whereas the unaffected carrier Father^+/−^ expressed primarily the wild-type allele (Figure 2j).

Neural migration can be quantified using a neurosphere migration assay, which measures radial migration of NPCs outward from a central neurosphere; aberrantly reduced migration correlates with a SZ diagnosis.^5^ The SZ Daughter^+/−^ had significantly decreased migration (255.6±55.3 μm) relative to five controls (512.0±111.2 μm) (*P*=0.0004) (Figure 2k and l); neither the unaffected carrier Father^+/−^ (551.5±56.1 μm) nor the non-carrier Mother^+/+^ (650.4±132.6 μm) showed aberrant migration. Migration and expression of exon 2–3 (*P*=0.3779) were not significantly correlated, but migration and expression of exons 14–15 (*r*=0.6959, *P*=0.0083) and exons 23–24 (*r*=−0.8230, *P*=0.003) were significantly correlated (Figure 2m). Finally, both preferential expression of the deleted *CNTNAP2* allele (Figure 2g,j) and significantly reduced neural migration (Figure 2k,l) occurred only in the SZ Daughter^+/−^.

This study, although necessarily preliminary owing to its observational nature and the inclusion of just one family trio, reveals insights into the complicated genetics underlying SZ, and warrants replication across additional family trios with discordantly inherited genetic lesions. Future studies will need to distinguish between at least three possibilities suggested by these data: (1) the carrier Father^+/−^ has protective alleles not inherited by the affected daughter; (2) the SZ Daughter^+/−^ has additional deleterious alleles, either *de novo* or inherited from her mother, not present in her father; (3) *CNTNAP2* structural deletions present with incomplete penetrance and variable expressivity, owing to the functional consequences of expressing variable levels of the mutated *CNTNAP2* allele. Our findings are consistent with a previous characterization of patient lymphocytes in an unrelated pedigree, in which a *CNTNAP2* autism spectrum disorder case exhibited significantly decreased *CNTNAP2* expression relative to the unaffected carrier mother (the carrier mother, in turn, showed significantly decreased *CNTNAP2* expression relative to the wild-type father and controls).^9^

In cases involving intragenic losses such as this one, abnormal alternative splicing and isoform dysregulation has already been posited to contribute to variable expressivity of *CNTNAP2* (reviewed in ref. 10). Moreover, preferential expression of either the mutant or wild-type allele is another possible explanation for the incomplete penetrance of SZ risk genes (reviewed in ref. 11); indeed a study of allele-biased expression in hiPSC-derived neurons identified putative SZ and autism spectrum disorder-associated genes, including *CNTNAP2*, to be robustly implicated in allele-biased expression.^12^ Although the mechanistic effectors remain unidentified, here we present evidence that differences in both exon- and allele-specific expression may have a critical role in SZ predisposition.

## Data deposition

All case and control hiPSCs will be deposited with the NIMH Center for Collaborative Studies of Mental Disorders at RUCDR. Reprints and permissions information are available at http://www.nature.com/npjschz.

## Figures and Tables

**Figure 1:**
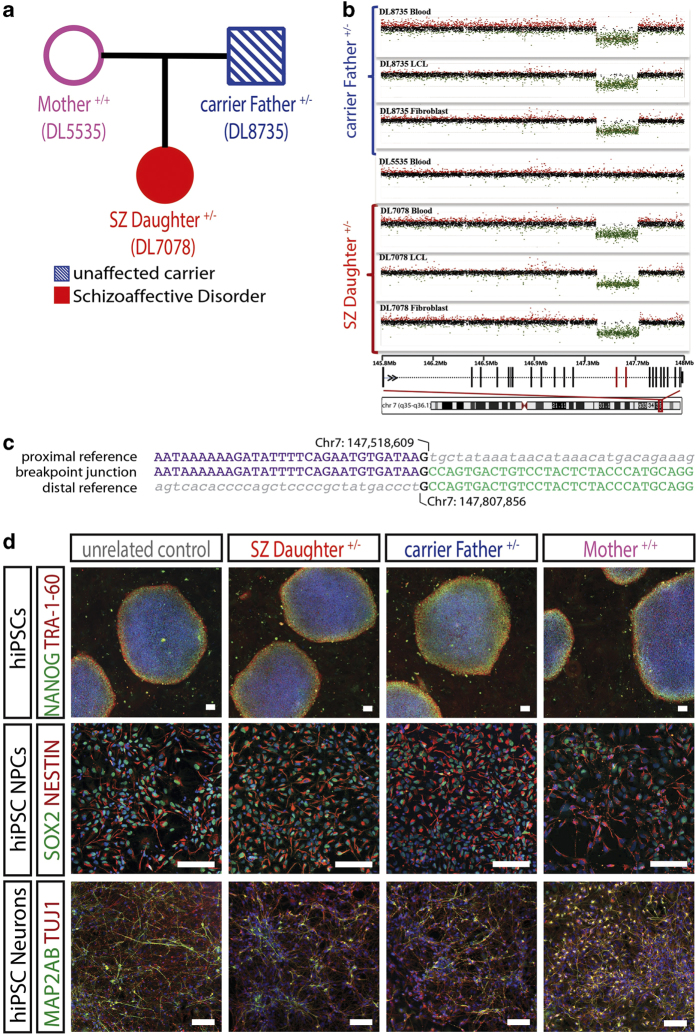
Description of *CNTNAP2* family trio and generation of subject-specific hiPSCs, NPCs, and neurons. (**a**) Pedigree of the family trio. The proband ‘SZ Daughter^+/−^’ has a heterozygous *CNTNAP2* deletion that was inherited from her unaffected carrier Father^+/−^. (**b**) CNV identification of chromosome 7 deletion (7q35q36.1) in *CNTNAP2* in unaffected carrier Father^+/−^ (**top**) and affected proband SZ Daughter^+/−^(**bottom**). (**c**) Schematic of *CNTNAP2* gene and mapping of breakpoint at chromosome 7 (147518609–147807856 hg19). (**d**) **Top**. hiPSCs express NANOG (green) and TRA-1-60 (red). DAPI (blue). ×40, bar=100 μm. **Middle**. hiPSC NPCs express NESTIN (red) and SOX2 (green). DAPI (blue). ×100, scale bar=100 μm. **Bottom**. hiPSC neurons express βIII-TUBULIN (red) and the dendritic marker MAP2AB (green). DAPI (blue). 100×, scale bar=100 μm. DAPI, 4′,6-diamidino-2-phenylindole.

**Figure 2:**
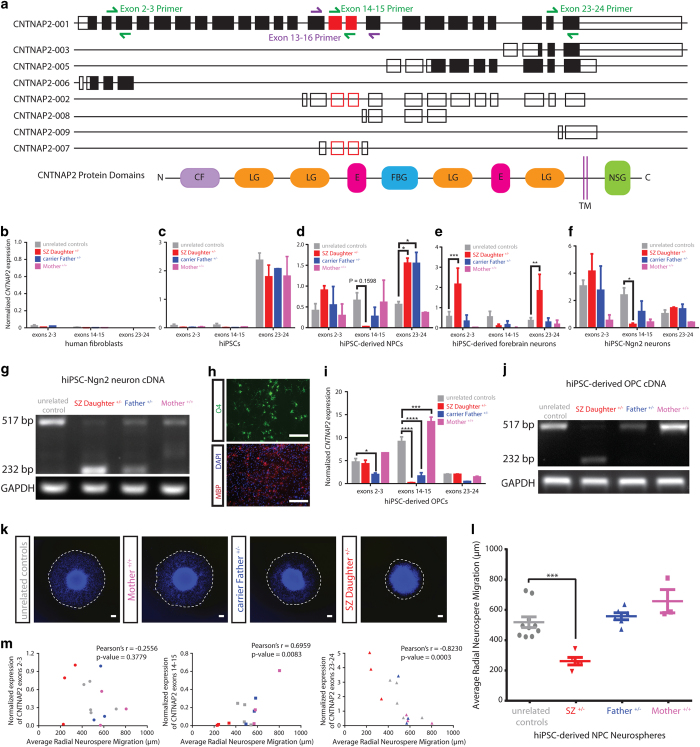
Exon- and allele-specific *CNTNAP2* expression in hiPSC-derived NPCs, neurons and OPCs correlates with aberrant migration in hiPSC forebrain NPCs. (**a**) Schematic illustrating splicing patterns of eight known *CNTNAP2* transcript variants as well as protein domains. *CNTNAP2* has eight transcript variants: four protein-coding transcripts (*CNTNAP2-001*, *CNTNAP2-003, CNTNAP2-005,* and *CNTNAP2-006*), three processed transcripts (*CNTNAP2-002*, *CNTNAP2-008,* and *CNTNAP2-009*), and one retained intron (*CNTNAP2-007)*. *CNTNAP2-001* is the full-length transcript comprised of 24 exons. Filled rectangles denote protein-coding regions, unfilled rectangles denote untranslated regions (5′ and 3′ UTRs; processed transcripts; retained introns); round rectangles denote protein domains. Red rectangles denote exons 14 and 15, which are missing in the deletion lines. Green primers denote those used in RT qPCR experiments; purple primers denote those used in allele-specific PCR. Exon-specific *CNTNAP2* expression in patient fibroblasts and hiPSCs. Exon-specific expression patterns (exons 2–3, 14–15, 23–24) of five unrelated controls, SZ Daughter^+/−^, carrier Father^+/−^, and unaffected Mother^+/+^ in primary human fibroblasts (**b**) and hiPSCs (**c**). (**d**–**f**) Exon-specific expression patterns (exons 2–3, 14–15, 23–24) of five unrelated controls, SZ Daughter^+/−^, carrier Father^+/−^, and unaffected Mother^+/+^ in hiPSC-derived forebrain NPCs (**d**), 6-week-old hiPSC-derived forebrain neurons (**e**), and 2-week-old hiPSC-derived NPC *Ngn2*-induced neurons (**f**). (**g**) Allele-specific expression patterns of exons 13–16 in wild-type (517 bp) and deletion (232 bp) *CNTNAP2* alleles from patient-derived *Ngn2*-induced neurons. (**h**–**j**) Exon- and allele-specific *CNTNAP2* expression in hiPSC-derived OPCs. Representative images of hiPSC-derived OPCs. Top: live cells stained for OPC marker O4 shown in green. Bottom: immunohistochemical staining of fixed cells for myelin basic protein in red, DAPI-stained nuclei in blue. bar=200 μm (**h**). Exon-specific expression patterns (exons 2–3, 14–15, 23–24) of one hiPSC line unrelated control, SZ Daughter^+/−^, carrier Father^+/−^, and unaffected Mother^+/+^ in hiPSC-derived forebrain OPCs (**i**). Allele-specific expression patterns of exons 13–16 in wild type (517 bp) and deletion (232 bp) *CNTNAP2* alleles from patient-derived OPCs (**j**). (**k**–**m**) Aberrant migration in hiPSC forebrain NPCs correlates with diagnosis, not *CNTNAP2* genotype. Representative images of hiPSC forebrain NPC neurosphere outgrowth assay. The average distance between the radius of the inner neurosphere (dense aggregate of nuclei) and outer circumference of cells (white dashed line) was calculated. DAPI-stained nuclei (blue). ×20; bar=200 μm (**k**). Average radial neurosphere migration by *CNTNAP2* carrier and non-carrier hiPSC-derived forebrain NPCs. Each data point represents the average radial migration of eight neurospheres (**l**). Pearson’s correlation analysis of exon-specific *CNTNAP2* expression (exons 2–3, left; exons 14–15, center; exons 23–34, right) and average radial neurosphere migration. (One extreme outlier of *CNTNAP2* exon 14–15 expression was excluded; with its inclusion, *r*=0.5427, *P*=0.0049) (**m**) In all NPC and neuron experiments, results represent averaged results from three hiPSC lines each derived from the non-carrier Mother^+/+^, the unaffected carrier Father^+/−^, and the SZ Daughter^+/−^, as well as one hiPSC line from each of five unrelated controls. OPC experiments represent averaged results from two independent differentiations of OPCs from one hiPSC line each, derived from the non-carrier Mother^+/+^, the unaffected carrier Father^+/−^, and the SZ Daughter^+/−^, as well as one hiPSC line from one unrelated control. CF, coagulation factor 5/8 C-terminal type domain; LG, laminin G domain; E, epidermal growth factor-like domain; FBG, fibrinogen-like domain; NSG, neurexin/syndecan/glycophorin C domain; TM, transmembrane domain B-C. Error bars are s.e.; *P<0.05, **P<0.01, ***P<0.001, ****P<0.0001. DAPI, 4′,6-diamidino-2-phenylindole.
