# Supplementary material for: Characterization of molecular and cellular phenotypes associated with a heterozygous CNTNAP2 deletion using patient-derived hiPSC neural cells
Source: NPJ Schizophr. 2015 Jun 24;1:15019–. doi: 10.1038/npjschz.2015.19 (PMC4789165; doi:10.1038/npjschz.2015.19)
Supplement: Supplementary Information [file npjschz201519-s1.doc]

### SUPPLEMENTAL METHODS

*Description of SZ patients*

The proband met criteria for a DSM-IV diagnosis of schizo-affective disorder, depressed subtype based on a Structured Clinical Interview for DSM-IV (SCID) and review of hospital records. Neither parent met criteria for an Axis I disorder nor a SZ-related personality disorder based on a SCID interview and the Structured Interview for Schizotypal Symptoms, respectively. Written informed consent was obtained from all subjects. One hour before the procedure a topical analgesic was locally applied to the skin where the biopsy was obtained in order to minimize any discomfort from the procedure. The surface was cleansed and the area to be biopsied was injected with 1% xylocaine with epinephrine intradermally. A 4mm punch biopsy was taken; antibiotic ointment and a bandage were applied. The family trio (Caucasian) was biopsied at McLean Hospital. 4mm dermal punch biopsies were collected and shipped overnight at room temperature in HF media (DMEM (Life Technologies) + 20% FBS (Gemini) + anti/anti (Life Technologies)) to Icahn School of Medicine at Mount Sinai.

- The Rapoport laboratory at NIH generously provided control fibroblasts from unrelated individuals. The following (Caucasian) controls were used: NSB553 (male), NSB2607 (male), NSB3113 (female), NSB3121 (female), NSB3130 (male).

*Genotyping methods*

Initial genome scans were performed using NimbleGen HD2 arrays (NimbleGen Systems Inc.) according to the manufacturer’s instructions using a standard reference genome SKN1. NimbleGen HD2 dual-color intensity data were normalized in a two-step process: first, a “spatial” normalization of probes was performed to adjust for regional differences in intensities across the surface of the array; second, the Cy5 and Cy3 intensities were adjusted to a fitting curve by invariant set normalization, preserving the variability in the data. The log_2_ ratio for each probe was then estimated using the geometric mean of normalized and raw intensity data.

To further determine the size, genomic extent and gene content for each rearrangement, we designed a tiling-path oligonucleotide microarray spanning the 7q34-q36.1 chromosomal region. A custom Agilent Technologies (Santa Clara, CA) microarray was designed using the Agilent e-array website (http://earray.chem.agilent.com/earray/). We selected 20,000 probes covering chr7: 140,000,000-150,000,000 (hg19), which represents an average distribution of 1 probe every 500 bp. Probe labeling and hybridization were performed as described.

*Breakpoint junction sequencing*

Sample-specific primers for PCR were designed at the apparent boundaries of the deleted segment as inferred from the aCGH result marked by a transition from normal copy number to copy number loss. Long-range PCR reactions were performed using TaKaRa LA *Taq* (Clontech, Mountain View, CA). PCR products were sequenced by Sanger sequencing methodology.

*HF culture and hiPSC derivation*

Biopsies were digested at 37°C for 12 hours in enzyme digestion media (DMEM (Life Technologies) + 20% FBS (Gemini) + 1% collagenase (Life Technologies) + 1 unit/ml dispase (Sigma)), washed twice and plated in a minimal media volume of HF media on 0.1% gelatin coated plates. Media was changed every 72 hours. Fibroblasts grew from biopsy to confluency in 1-2 weeks and were then split 1:3 approximately every 7 days. HFs were cultured on plates treated with 0.1% gelatin (in milli‐Q water) for a minimum of 30 minutes and grown in HF media.

Replicating but recently confluent HFs were transfected once with Cytotune Sendai virus expressing OCT4, SOX2, KLF4 and cMYC (Life Technologies) and after 3-5 days of recovery, with TrypleE (Life Technologies) and re-plated onto a 10-cm dish containing 1 million mouse embryonic fibroblasts (mEFs). Cells were switched to HUES media (DMEM/F12 (Invitrogen), 20% KO-Serum Replacement (Invitrogen), 1x Glutamax (Invitrogen), 1x NEAA (Invitrogen), 1x 2‐mercaptoethanol (Sigma) and 20 ng/ml FGF2 (Invitrogen)) and fed every 2-3 days. hiPSC colonies were manually picked and clonally plated onto 24-well mEF plates. hiPSC lines were either maintained on mEFs in HUES media or on Matrigel (BD) in TeSR media (Stemcell Technologies). At early passages, hiPSCs were split through manual passaging. At higher passages, hiPSC could be enzymatically passaged with Collagenase (1mg/ml in DMEM) (Sigma). Cells were frozen in cold freezing media (HUES media, 10% DMSO). At approximately passage ten, karyotyping analysis was performed by Wicell Cytogenetics (Madison WI); only karyotypically normal lines were used for subsequent studies.

All hiPSC lines were validated by long-term expansion beyond ten passages, immunohistochemistry for pluripotency markers (NANOG, TRA-1-60, OCT4 and SOX2), and normal karyotype.

###### hiPSC differentiation to NPCs

hiPSCs grown in HUES media on mEFs were incubated with Collagenase (1 mg/ml in DMEM) at 37°C for one to two hours until colonies lifted from the plate and were transferred to a nonadherent plate (Corning). Embryoid Bodies (EBs) were grown in suspension with dual-SMAD inhibition (0.1mM LDN193189 (Stemgent) and 10mM SB431542 (Tocris)) N2/B27 media (DMEM/F12-Glutamax (Invitrogen), 1x N2 (Invitrogen), 1X B27 (Invitrogen)). After seven days, EBs were plated in N2/B27 media with 1 μg/ml Laminin (Invitrogen) onto poly-ornithine /Laminin-coated plates. Visible rosettes formed within one week, were cultured in NPC media (DMEM/F12, 1x N2, 1x B27-RA (Invitrogen), 1 μg/ml Laminin and 20 ng/ml FGF2) and dissociated after one-week using Neural Rosette Selection Reagent (STEMdiff^TM^) for 60 minutes at 37°C before being plated in NPC media on poly-ornithine/Laminin-coated plates. NPCs were maintained at high density, grown on poly-ornithine /Laminin-coated plates in NPC media and split approximately 1:4 every week with Accutase (Millipore).

NPCs were validated by both immunohistochemical confirmation that the majority of cells were positive for SOX2 and NESTIN, as well as their propensity to robustly yield populations comprised of >70% βIII-TUBULIN-positive neurons.

Forebrain NPCs were maintained at high density, grown on either Matrigel or poly-ornithine /Laminin-coated plates in NPC media (DMEM/F12, 1x N2, 1x B27-RA (Invitrogen), 1 μg/ml Laminin (Invitrogen) and 20 ng/ml FGF2 (Invitrogen) and split approximately 1:3-1:4 every week with Accutase (Millipore). NPCs could be expanded up to approximately 10 passages. Experiments represent averaged results from NPCs differentiated from three hiPSC lines each, derived from the non-carrier Mother^+/+^, the unaffected carrier Father^+/-^ and the SZ Daughter^+/-^, as well as one hiPSC line from each of five unrelated controls.

*Directed neuronal differentiation*

For neuronal differentiations, NPCs were dissociated with Accutase (Millipore) and plated in neuron media (DMEM/F12-Glutamax, 1x N2, 1X B27-RA, 20 ng/ml BDNF (Peprotech), 20 ng/ml GDNF (Peprotech), 1 mM dibutyrl-cyclicAMP (Sigma), 1 μg/ml Laminin (Invitrogen), and 200 nM ascorbic acid (Sigma) onto poly-ornthine/Laminin-coated plates. Neuron media was changed every week. Directed neuronal differentiation neurons were maintained for 6 weeks at 37°C until harvest or staining. Experiments represent averaged results from 6-week-old neurons differentiated from three hiPSC lines each derived from the non-carrier Mother^+/+^, the unaffected carrier Father^+/-^ and the SZ Daughter^+/-^ as well as one hiPSC line from each of five unrelated controls.

*Ngn2-induced neuronal differentiation*

For *Ngn2*-induced neuronal differentiation, NPCs were dissociated with Accutase (Millipore) and plated at a density of 1.5 million cells per well of a 6-well plate in NPC media onto poly-ornithine/Laminin-coated plates. 24 hours after plating, the media was replaced, lentiviruses were added (a TetO-mNgn2-T2A-PuroR together with a constitutive reverse tetracycline transactivator (rtTA), both in third generation lentiviral vectors), and plates were spinfected for 1 hour at 1000g. NPCs were incubated at 37°C for an additional 3-4 hours before viruses were removed and media replaced with fresh NPC media. 24 hours after spinfection, NPC media was supplemented with doxycycline (1 µg/ml; Sigma) to activate TetO-mNgn2-T2A-PuroR expression, then after 24 hours of transgene induction, puromycin (1 µg/ml; Sigma) was added to the media for 24 hours of selection. Finally, media was switched to neuron media and neurons were allowed to mature for fourteen days; for the first six days only neuron media was supplemented with doxycycline; half-media changes were performed every other day. Experiments represent averaged results from Ngn2-induced neurons from three hiPSC lines each derived from the non-carrier Mother^+/+^, the unaffected carrier Father^+/-^ and the SZ Daughter^+/-^ as well as one hiPSC line from each of five unrelated controls.

*Oligodendrocyte differentiation*

hiPSCs were plated on Matrigel (BD Biosciences) in mTeSR1 medium (StemCell Technologies) containing 10µM Rock Inhibitor (Y-27632; StemGent) for 24 hours. Cells were induced to neural differentiation at day 0 by using mTeSR Custom lacking Lithium Chloride, GABA, Pipecolic Acid, bFGF and TGFβ1 (StemCell Technologies), supplemented with 100nM all-trans-retinoic acid (RA; Sigma) and the small molecules SB431542 10µM (StemGent) and LDN193189 250nM (StemGent). Media changes occurred daily until d8, when the media was switched to N2 medium (DMEM/F12, N2 supplement) containing 100nM RA, 1µM SAG (Millipore). Media was changed daily and at d12 cells were detached using a cell-lifter and re-plated into Ultra-low attachment plates in N2B27 medium (DMEM/F12, N2 supplement, B27 supplement, insulin 25µg/ml) containing 100nM RA and 1µM SAG. At day 20, medium was switched to PDGF medium (N2B27 Medium with PDGFaa 10ng/ml, IGF-1 10ng/ml, HGF 5ng/ml, NT3 10ng/ml, T3 60 ng/ml, biotin 100ng/ml, cAMP 1µM) and changed every other day. At day 30, spheres were plated onto plates coated with poly-L-ornithine hydrobromide (50µg/ml; Sigma) and Laminin (20µg/ml; Life Technologies) in a 2 spheres/cm^2^ density and medium was switched to Glial Medium (N2B27 Medium with T3 60ng/ml, biotin 100ng/ml, cAMP 1µM, HEPES 10mM, Ascorbic Acid 20µg/ml). Media changes occurred every other day until day 64 when cultures were stopped for staining and RNA extraction. Experiments represent averaged results from two independent differentiations of OPCs from one hiPSC line each, derived from the non-carrier Mother^+/+^, the unaffected carrier Father^+/-^ and the SZ Daughter^+/-^, as well as one hiPSC line from one unrelated control.

*RT qPCR experiments*

Gene expression analysis was performed on fibroblasts, hiPSCs, hiPSC-derived NPCs, hiPSC-derived neurons, and Ngn2-induced neurons. Cells were lysed in RNA BEE (AMS Biotechnology). RNA was chloroform extracted, pelleted with isopropanol, washed with 70% ethanol and re-suspended in DEPC-treated water. RNA was treated with TURBO DNA-free^TM^ (Life Technologies) for 30 minutes at 37°C and then the reaction was inactivated by incubation with DNase Inactivation Reagent at R.T. for 5 minutes. DNase Inactivation Reagent was then pelleted by centrifugation at 10,000 x g for 1.5 minutes, and the RNA was transferred to a fresh tube. RNA concentration and 260/280 ratio were measured by NanoDrop™ Lite Spectrophotometer (Thermo Scientific). cDNA was synthesized using SuperScript® III First-Strand Synthesis SuperMix for qRT-PCR. Reverse transcription was performed at 50°C for 30 minutes, inactivated for 5 minutes at 85°C and then treated with RNase H for 20 minutes at 37°C. qPCR was performed with Fast SYBR® Green Master Mix (Life Technologies) on 7900HT Fast Real-Time PCR System (Applied Biosystems) using validated primers that were designed on Primer-BLAST. Three primer pairs were designed and tested for each primer pair that was reported; of the three pairs, the pair with the best efficiency and R^2^ value was employed for all subsequent RT qPCR experiments. All validated primers (sequences listed in **SI Table 1)** had: i) R^2^ value of ≥ 0.997, ii) efficiency between 90-110%, iii) a single peak in melt curve analysis, indicating that a single amplicon was generated by qPCR. *CNTNAP2* expression was normalized to average of two housekeeping genes, *ACTIN* and *GAPDH*. All qPCR experiments were performed in triplicate; moreover, with the exception of tests of primary fibroblasts, experiments represent averaged results from three hiPSC lines each derived from the non-carrier Mother^+/+^, the unaffected carrier Father^+/-^ and the SZ Daughter^+/-^ as well as one hiPSC line from each of five unrelated controls.

According to Ensembl Genome Browser, *CNTNAP2* has eight splice variants; four protein-coding transcripts *(CNTNAP2-001, CNTNAP2-003, CNTNAP2-005, and CNTNAP2-006*), three processed transcripts (*CNTNAP2-002, CNTNAP2-008, and CNTNAP2-009*), and one retained intron (*CNTNAP2-007).* *CNTNAP2-001* is the full-length transcript comprised of 24 exons. Exons 2-4 comprise a coagulation factor 5/8 C-terminal type domain, exon 8-9 comprise fibrinogen-like domain, and exons 10-11 and exon 16 encode two epidermal growth factor-like domains. In addition, there are four laminin G domains (distributed in exons 5-7, 8-10, 6-18 and 19-22 of the full-length transcript), as well as one neurexin/syndecan/glycophorin C in exon 24. Primers targeting three regions of exons of *CNTNAP2* were designed (**SI Table 1**): pair one spans exons 2-3, pair two spans exons 14-15, and pair three spans exons 23-24. Primer pair 1 targets *CNTNAP2-001* and *CNTNAP2-006*; pair 2 targets *CNTNAP2-001, CNTNAP2-002,* and *CNTNAP2-007*; pair 3 targets *CNTNAP2-001, CNTNAP2-003, CNTNAP2-005,* and *CNTNAP2-002*.

*Allele-specific PCR*

For allele-specific PCR, *Ngn2*-induced neuron cDNA was used as a template to amplify *CNTNAP2* exon 13-16. Primer sequences are listed in SI Table 1. Using Phusion® High-Fidelity DNA Polymerase (NEB), PCR steps consisted of the following: 30 seconds at 98°C; 35 cycles of 10 seconds at 98°C, 20 seconds at 60°C, and 20 seconds at 72°C; and 5 minutes at 4°C for final extension. The final PCR product was separated on 2% agarose gel, and images of the bands corresponding to the expected sizes (517bp and 232bp) taken by AlphaImager® Mini System (ProteinSimple).

*Immunohistochemistry*

Cells were fixed in 4% paraformaldehyde in PBS at 4°C for 10 minutes. hiPSCs and NPCs were permeabilized at room temperature for 15 minutes in 1.0% Triton in PBS. All cells were blocked in 5% donkey serum with 0.1% Triton at room temperature for 30 minutes. The following primary antibodies and dilutions were used: 1:200; goat anti-Nanog (R&D), 1:200; mouse anti-Tra-1-60 (Millipore), 1:200; goat anti-Sox2 (Santa Cruz), 1:200; rabbit anti-Nestin (Millipore),1:400; rabbit anti-βIII-tubulin (Covance), and 1:200; mouse anti-MAP2ABs (Sigma). Secondary antibodies were Alexa donkey 555 anti-rabbit (Life Technologies), Alexa donkey 488 and 555 anti-mouse (Life Technologies), and Alexa donkey 488 anti-goat (Life Technologies); all were used at 1:200. To visualize nuclei, cells were stained with 0.5 μg/ml DAPI (4',6-diamidino-2-phenylindole). Images were acquired using an Olympus IX51 Fluorescence Microscope or a Zeiss LSM780 confocal microscope.

*Neurosphere migration assay*

NPCs were dissociated with accutase and then cultured for 48-96 hours in nonadherent plates to generate neurospheres. Neurospheres were manually picked and cultured in Matrigel matrix (0.05 mg/ml). 0.1 ml of Matrigel was plated in cold NPC media on a 96-well plate 1 hour prior to neurosphere plating; following neurosphere picking, an additional 0.5 mg Matrigel was added in cold NPC media per 96-well plate). Average radial migration from each neurosphere was measured using NIH ImageJ. Each data point presented represents the average radial migration of eight neurospheres.

*Statistical analysis*

In all NPC and neuron experiments, results represent averaged results from three hiPSC lines each derived from the non-carrier Mother^+/+^, the unaffected carrier Father^+/-^ and the SZ Daughter^+/-^, as well as one hiPSC line from each of five unrelated controls. OPC experiments represent averaged results from two independent differentiations of OPCs from one hiPSC line each, derived from the non-carrier Mother^+/+^, the unaffected carrier Father^+/-^ and the SZ Daughter^+/-^, as well as one hiPSC line from one unrelated control.

For gene expression analyses, two-way ANOVA followed by Dunnett’s multiple comparisons test was performed. For neurosphere migration assay, one-way ANOVA followed by Tukey’s multiple comparisons test was performed. For correlation analyses, Pearson’s correlation coefficients were computed. All statistical analyses were performed using GraphPad Prism version 6.01 for Windows, GraphPad Software, La Jolla California USA, www.graphpad.com. In all figures (except for OPCs, which consist of one hiPSC line from one unrelated control and one hiPSC line from each member of the trio), unrelated control data are from one iPSC line from each of the five unrelated controls, whereas trio data are from three hiPSC line from each member of the trio. Error bars represent standard error unless otherwise noted. *P<0.05, **P< 0.01, ***P< 0.001, ****P< 0.0001.

| **SI Table 1. Primer sequence list for RT qPCR experiments** | | |
| --- | --- | --- |
| **Gene** | **Forward primer sequence** | **Reverse primer sequence** |
| *ACTIN* | 5’–AAACTGGAACGGTGAAGGTG–3’ | 5’–AGAGAAGTGGGGTGGCTTTT–3’ |
| *GAPDH* | 5’–TGTTGCCATCAATGACCCCTT–3’ | 5’–CTCCACGACGTACTCAGCG–3’ |
| *CNTNAP2* Exon 2-3 | 5’–GCTATTCTCCCGGCTATGCCA–3’ | 5’–GTCGCTGTCTGATGGAGACCA–3’ |
| *CNTNAP2* Exon 14-15 | 5’–ACGCGGACTACAAGCAATGG–3’ | 5’–ATCTCCAACCACCACTTGGC–3’ |
| *CNTNAP2* Exon 23-24 | 5’–TAATCCAGGACAAGGCCAAGC–3’ | 5’–CACAGCAATGACGCCTCCAA–3’ |
| *CNTNAP2* Exon 13-16 | 5’–GGTCGGCTACAACCCAGAAA–3’ | 5’–GTCAGCGCTAGTTTCCCCTT–3’ |
